# A Fatal Omen

**Published:** 1900-03-31

**Authors:** 


### A FATAL OMEN.

It is always well to be aware of any sign or symptom which has been shown by experience to have a special prognostic significance, however little one may understand the connection between the event and its forerunner. We would therefore draw attention to a remark made by Dr. Lee Dickenson at the last meeting of the Pathological Society in regard to the significance of albumosuria. Albumose in the urine was, he said, distinguished from all other proteids by its coagulation at a low temperature, and by the fact that it redissolved at a higher temperature. These albumoses were not only found in cases of bone disease, but occurred also in certain cases of glycosuria, myxœdema, and leucocythæmia, and he considered that the finding of albumose in the urine was almost always a fatal omen. This is a matter which should be carefully watched, for omens, if of any constancy, are often of much value in forming a prognosis.

